# *KMT2A* Rearrangements in Leukemias: Molecular Aspects and Therapeutic Perspectives

**DOI:** 10.3390/ijms25169023

**Published:** 2024-08-20

**Authors:** Luca Guarnera, Matteo D’Addona, Carlos Bravo-Perez, Valeria Visconte

**Affiliations:** 1 Department of Translational Hematology & Oncology Research, Taussig Cancer Institute, Cleveland Clinic, Cleveland, OH 44114, USA; guarnel@ccf.org (L.G.); daddonm2@ccf.org (M.D.); bravoc2@ccf.org (C.B.-P.); 2 Department of Biomedicine and Prevention, Tor Vergata University of Rome, 00133 Rome, Italy; 3 Department of Hematology and Medical Oncology, Hospital Universitario Morales Meseguer, CIBERER—Instituto de Salud Carlos III, University of Murcia, IMIB-Pascual Parrilla, 30005 Murcia, Spain

**Keywords:** *KMT2A* rearrangements, molecular lesions, therapies

## Abstract

*KMT2A* (alias: mixed-lineage leukemia [*MLL*]) gene mapping on chromosome 11q23 encodes the lysine-specific histone N-methyltransferase 2A and promotes transcription by inducing an open chromatin conformation. Numerous genomic breakpoints within the *KMT2A* gene have been reported in young children and adults with hematologic disorders and are present in up to 10% of acute leukemias. These rearrangements describe distinct features and worse prognosis depending on the fusion partner, characterized by chemotherapy resistance and high rates of relapse, with a progression-free survival of 30–40% and overall survival below 25%. Less intensive regimens are used in pediatric patients, while new combination therapies and targeted immunotherapeutic agents are being explored in adults. Beneficial therapeutic effects, and even cure, can be reached with hematopoietic stem cell transplantation, mainly in young children with dismal molecular lesions; however, delayed related toxicities represent a concern. Herein, we summarize the translocation partner genes and partial tandem duplications of the *KMT2A* gene, their molecular impact, clinical aspects, and novel targeted therapies.

## 1. Introduction

**Figure 1:**
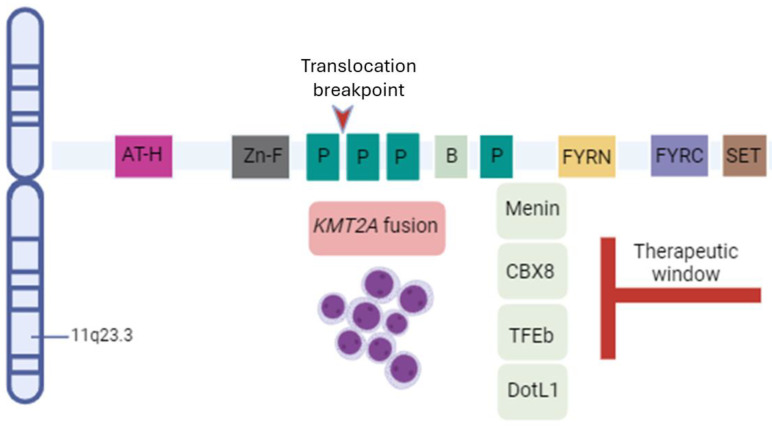
Schematic representation of *KTM2A* gene structure and cofactors. *KMT2A* gene is located on chromosome 11q23.3. Presence of *KMT2A* fusion defines some subtypes of leukemia. Fusion proteins recruit transcription cofactors to promote transcription elongation. A potential therapeutic window derives from the inhibition of such cofactors (for instance, menin). Abbreviations: AT-H, AT-hooks; Zn-F, zinc fingers; P, PHD fingers; B, bromodomain; FYRN, FY-rich domain N-terminal; FYRC, FY-rich domain C-terminal.

Lysine-specific N-methyltransferase 2A (KMT2A), also known as mixed-lineage leukemia (MLL), is the target of balanced rearrangements. Two breakpoint cluster regions have been identified: a major region between intron 7 and exon 13, and a minor region between intron 20 and exon 24. These rearrangements are very diverse and, due to the heterogeneous nature of the fusion partners, therapeutic decision is difficult. Patients with these genomic lesions are characterized by chemotherapy resistance and high rates of relapse after standard therapies. The genomic sequencing of breakpoints has also been used to monitor measurable residual disease [1]. Typically, these rearrangements are associated with poor prognosis, likely due to the marked epigenetic changes induced by the lesions, with fusions displaying an abnormal increase in H3K79 methylation. The rearrangements usually impact transcriptional regulatory genes, including homeobox A cluster (HOXA), which are linked to leukemia. Recently, acute myeloid leukemia (AML) with rearrangement of the nucleoporin 98 (*NUP98*) gene has been found to be sensitive to menin inhibitors, which hold promise to play an important role in this adverse disease subgroup (Figure 1).

This review provides an analysis of all *KMT2A* rearrangements in AML patients, describing their specific prognostic outcomes in different age ranges. We conducted an extensive narrative review of the literature, focusing on clinical approaches among the chemotherapies available, the role of transplant, and new therapeutic strategies using menin inhibitors.

## 2. Biology of Rearrangements

Rearrangements involving *KMT2A* and its fusion partner genes are found in precursor B-cell acute lymphoblastic leukemia (B-ALL), T-ALL, AML, myelodysplastic neoplasm (MDS), mixed-lineage (biphenotypic) leukemia (MPAL), and secondary leukemia [1,2,3]. Using the next-generation sequencing (NGS) of primary AML specimens, Lavallée and colleagues identified the first unifying genetic network of *KMT2A*. Two subgroups of *KMT2A*-rearranged leukemia have been described: ones characterized by translocations with other genes, and one characterized by partial tandem duplications (PTDs) [4].

The first one accounts for 5−10% of adult AML patients [5], while PTD rearrangements are found in an additional 5–6% of AML patients [6]. Study done by Meyer et al. in 2023 showed data obtained from 3401 acute leukemia patients using LDI-PCR technology and targeted NGS [1]. All the identified rearrangements were characterized by many reciprocal chromosomal translocations and, to a lesser extent, also by spliced fusions, inversions, deletions, insertions, and PTD. AML patients displayed a more heterogenous landscape of partner genes (Table 1). Among them, the most frequent one is *MLLT3* (30.4%). About 84.8% of all AML patients are characterized by seven fusion genes: *KMT2A::MLLT3*, *KMT2A::MLLT10*, *KMT2A-PTD*, *KMT2A::ELL*, *KMT2A::AFDN*, *KMT2A::MLLT1*, and *KMT2A::MLLT11*. To improve the accurate identification of complex *KMT2A* gene rearrangements, considerations could be given to the incorporation of multiplex ligation probe amplification (MLPA), optical genome mapping, or third-generation long-read sequencing into conventional molecular diagnostic algorithms based on short-read NGS [7,8,9].

Regarding the breakpoint distribution of the *KMT2A* recombinome, this configuration almost invariably occurs in the major breakpoint cluster region 1 (*BCR1*) mapping between *KMT2A* intron 7 and *KMT2A* exon 13.

In terms of molecular associations, co-mutational analysis reveals a lower mutational rate than AML with a normal karyotype, with an enrichment of mutations in genes of the RAS pathway (e.g., *KRAS*, *NRAS*, and *PTPN11*, up to 30%) and *FLT3* (8–14%). *KRAS* mutations occurred more often in patients with t(6;11) (q27;q23)/*KMT2A::AFDN* compared with patients with other 11q23/*KMT2A* subsets [10].

This holds also true for *KMT2A::EDC4*, a new breakpoint fusion gene spanning exon 7 of *KMT2A* and exon 5 of *EDC4* gene, involving DNA damage response and repair [11]. Along with the involvement of *KRAS* mutations, some studies have shown that subclonal-activating mutations, for instance *FLT3*^N676K^, accelerate *KMT2A-MLLT3* leukemia onset [12]. In relation to *KMT2A::AFF1* (*MLL::AF4*) fusions, it has been reported that specific alterations in circRNA expression might be oncogenic or sustain the oncogenic properties of chimeric proteins. However, these findings require further investigation [13].

## 3. Clinical Aspects

Overall, *KMT2A* rearrangements are present in up to 10% of acute leukemias. However, the array of disease subtypes embodying this genetic abnormality is remarkable, and distinctive clinical profiles can be identified. According to the age at presentation, *KMT2A*-rearranged leukemias represent 80% of cases in infants (<1 of age) and 5–15% of cases during childhood and adulthood, including ALL, AML, MPAL, and MDS/secondary AML [14,15,16,17]. As specific diagnostic entities with recurrent genetic abnormalities, B-ALL/lymphoblastic lymphoma and AML with *KMT2A* rearrangements are categories included in the latest classification of the World Health Organization (5th WHO) and the International Consensus Classification (2022 ICC) [18,19,20]. Nevertheless, regardless of disease subtype, the prognosis of *KMT2A*-rearranged leukemias is generally poor, with a progression-free survival (PFS) of 30–40% and an overall survival (OS) below 25% [14,15,16,17]. 

### 3.1. Incidence, Clinical Features, and Prognosis in Infancy and Childhood

There is a dramatic incidence of *KMT2A* rearrangements in infants, constituting the driver genetic alteration in 80% of the acute leukemias during this period. Most of them are B-ALL (80–90%), followed by T-ALL, AML (up to 5–10% each), and other disease subtypes (<5%) [21]. Molecularly, the most frequent fusion partners of *KMT2A* are *AFF1*, *MLLT1*, and *MLLT3* [1]. Infant-onset B-ALL with *KMT2A* rearrangement normally exerts a very immature phenotype (pro-B), with the frequent aberrant co-expression of myeloid markers. Patients normally present with hyperleukocytosis and extramedullary disease with central nervous system involvement and have a poor prognosis [22]. The other 20% of infant ALL cases that were not *KMT2A*-rearranged, also known as *KMT2A*-germline since the gene remained in its unaltered (germline) configuration, were found to carry somatic rearrangements in other oncogenes. Genetic analysis showed an enrichment in the *NUTM1* and *PAX5* rearrangements. Clinically, *KMT2A*-germline cases are normally B-ALLs with a more mature immunophenotype. Genetic correlation studies have revealed that *NUTM1* rearrangements have an unusual favorable prognosis as compared to the rest of the infant ALL (PFS of 75–95%), while *PAX5* and other gene rearrangements have a high risk of relapse [23,24,25,26].

### 3.2. Incidence, Clinical Features, and Prognosis in Adulthood

During the juvenile period/adulthood, the incidence of *KMT2A* rearrangement significantly decreases, and at 50–60 years of age slightly increases again. Overall, it is present in 5–15% of leukemias. Both in children and adults, 50–60% of *KMT2A*-rearranged cases are ALL, 40% are AML, and <10% are other disease subtypes. Overall, the top three frequent fusion partners are again *AFF1*, *MLLT1*, and *MLLT3* [1]. However, they significantly differ between ALL and AML. In *KMT2A*-rearranged AML, the most prevalent fusion partner is *MLLT3*, followed by *MLLT10*, *AFDN*, *ELL*, *MLLT1*, and *MLLT11*. Additionally, in adults, *KMT2A-PTD* is a characteristic lesion that is present in up to 10% of cases [1].

Clinically, *KMT2A*-rearranged AML patients often present with high blast counts and monocytic or myelomonocytic differentiation. Analysis across fusions reveals that *KMT2A::MLLT3* and *KMT2A::MLLT10* usually have lower blast counts versus the other cases and, particularly in children, megakaryoblastic differentiation. *KMT2A*-rearranged AML is clearly associated with inferior outcomes in several clinical series, including refractory disease, poor PFS and OS, and higher risks of coagulopathy, bleeding, and early death than AML with a normal karyotype [10,27].

The analysis of large cohorts integrating molecular data suggests that fusion partners are of prognostic value, even though some studies do not confirm differences between them. *KMT2A::MLLT3* vs. other fusions seems to be associated with relatively better survival outcomes, both in children (PFS 50% vs. 14–46% in other fusions; OS: 63% vs. 22–47% in other fusions) and in adults (PFS 47% vs. 13% in other fusions; OS: 46% vs. 11% in other fusions) [16,27,28]. *KMT2A::AFDN* and *KMT2A::MLLT10* are among the rearrangements with the poorest prognosis, with an OS of 30% in children and 5–30% in adults. In a large series of pediatric patients, *KMT2A::MLLT11* showed a significantly more favorable outcome, with a PFS of 92% and OS of 100% [16]. Based on this, the 2017 European Leukemia Net (2017ELN) stratified *KMT2A* rearrangements into *KMT2A::MLLT3*, of intermediate prognosis, and other fusions, of adverse prognosis [29,30]. Similarly, the 2022ICC, within AML and related entities, incorporated different categories of *KMT2A*-rearranged AML, including AML with *KMT2A::MLLT3*, AML with other *KMT2A* rearrangements, and MPAL with *KMT2A* rearranged [20].

Other conventional factors determining clinical outcomes in *KMT2A*-rearranged AML are diverse and include the diagnosis of therapy-related disease, primary chemoresistance (adverse factor), age at diagnosis < 60 years, frontline treatment, and allogeneic hematopoietic stem cell transplantation (HSCT) after first remission (favorable factor) [27]. Different studies have explored the role of co-mutations in refining the aggressiveness of the disease without finding a definitive predictive effect, probably because of the heterogeneity of these studies [10,27].

Distinctive clinical associations of *KMT2A* rearrangements in myeloid neoplasms rather than de novo or relapsed AML also include lymphoid-to-myeloid lineage switching among B-ALL patients treated with B-cell-targeted immunotherapy (up to 60% with *KMT2A* rearrangements); [29] therapy-related myeloid neoplasms, in particular in patients treated with topoisomerase II inhibitors [6,30]; and MDS/secondary AML, in which *KMT2A-PTD* has been described in up to 7–10% of cases [30]. All these entities, despite having clinical, genetic, and mechanistic idiosyncrasies, are unified by a dismal prognosis [30]

## 4. Clinical Management of *KMT2A*-Rearranged AML and Current Clinical Practice

In recent years, new, promising, chemo-free approaches have been tested, with mixed outcomes in AML and MDS, enlarging therapeutic opportunities and allowing for tailored treatment strategies [31]. In this area, due to the not-negligible incidence and prognostic weight of *KMT2A*-rearranged AML, several drugs and strategies targeting this entity have been investigated.

Currently there is no specific therapy approved to target *KMT2A* rearrangements and, in clinical practice, treatment strategies are based on canonical risk stratification [32].

### 4.1. Chemotherapy

Bill et al. [10] described 172 patients with *KMT2A*-rearranged AML, classified as intermediate-risk *KMT2A::MLLT3* or as having adverse-risk rearrangements different from *KMT2A::MLLT3*, according to the ELN2017 classification [33]. The patients were similarly treated with Cancer and Leukemia Group B (CALGB)/Alliance for Clinical Trials in Oncology (Alliance) protocols. Overall, 68% of patients obtained complete remission (CR), with a 3-year disease-free survival (DFS) of 29% and an OS of 26%. Patients harboring *KMT2A::MLLT3* presented the highest OS (3-year OS 41%) and DFS (3-year DFS 47%) when compared to other *KMT2A* rearrangements. Pollard et al., in a sub-analysis of the Children’s Oncology Group (COG) Trial AAML0531, investigated the administration of Gemtuzumab Ozogamicin (GO), an anti-CD33 antibody–calicheamicin conjugate, in combination with conventional chemotherapy; 215 pediatric patients with *KMT2A*-rearranged AML showed an advantage in terms of 5-year OS and 5-year event-free survival (EFS) as a result of receiving GO (63% vs. 53%, and 48% vs. 29%, respectively). Furthermore, patients receiving CR showed lower relapse rates in the GO arm (40% vs. 66%) and improved 5-year DFS (57% vs. 33%). The advantage was consistent across risk classes and GO administration was independently associated with improved EFS, DFS, and reduced relapse rates [34]. However, these results were not confirmed in the adult population by the UK group by Dillon et al., who found no clear benefit of GO in this subset of patients [35]. 

### 4.2. Less Intensive Regimens

Ball et al. investigated the impact of venetoclax in combination with hypomethylating agents. The overall response rate was 83% in newly diagnosed AML (12 patients) and 17% in relapsed/refractory AML (12 patients), with a median OS of 11 months in the first group and 6 months in the second one [36]. 

### 4.3. Hematopoietic Stem Cell Transplantation

Several authors have explored the role of HSCT in *KMT2A*-rearranged AML. In 2015, a study by the Acute Leukemia Working Party EBMT showed that 2- and 3-year OS and DFS were 56% and 51%, and 51% and 47%, respectively. Furthermore, *KMT2A::MLLT3* rearrangement was confirmed to be the most common and the one with better prognosis [37]. Recently, Jiang et al. reported similar results analyzing a cohort of 42 patients, with a 2-year OS and DFS of 59.1% and 49.6%, respectively [38]. In a pediatric setting, Miyamura et al. observed a 3-year OS and DFS of 52.1% and 46.7%, respectively [39]. Menghrajani et al. analyzed a large population of *KMT2A*-rearranged AML patients undergoing HSCT and observed rates of OS and EFS similar to those of adverse-risk AML with no significant differences across the rearrangement partners [40]. Tong et al. focused on a small cohort of 22 patients with *KMT2A::MLLT3* rearrangement in CR undergoing umbilical-cord HSCT and observed a 3-year OS and DFS of 71.3% and 60.8%, respectively (significantly higher than in patients not undergoing HSCT) [41]. Finally, Antherieu et al. analyzed a cohort of *KMT2A-PTD* AML patients and found that those receiving HSCT presented a significantly improved outcome. In the multivariable analysis, HSCT and *FLT3*-ITD status were the only independent variables associated with OS [42]. 

## 5. Menin Inhibitors

Menin inhibitors find their biological background in the interaction between *KMT2A* and menin. From the identification of this pathogenetic mechanism in 2005 by Yokoyama et al. [43], ten years passed to the development of the first menin inhibitors, MI-463 and MI-503, developed by Borkin and colleagues in 2015 and successfully tested in AML mouse models harboring *MLL* rearrangements, granting a survival benefit without impairing normal hemopoiesis [44]. These results were confirmed in patient-derived xenografts treated with two small molecules (VTP50469 and MI-3454) belonging to the same class [45,46], showing an on-target mechanism of action and strong antileukemic activity, prolonging survival without toxicity.

### 5.1. Revumenib

The first, recently published, phase I clinical trial (AUGMENT-101 (NCT04065399)) on revumenib (previously known as SNDX-5613), a selective oral inhibitor of the menin–KMT2A interaction, investigated the effect of this drug in R/R AML. In total, 68 heavily pre-treated (median of four previous lines of therapy) patients were enrolled, of whom 46 had *KMT2A* rearrangements. Grade III or higher treatment-related adverse events were observed in 16% of patients and 16% patients presented differentiation syndrome (all grade II). The trial protocol included an RNA-seq analysis that highlighted a downregulation of the critical leukemogenic targets MEIS1, homeobox A9 (HOXA9), pre-B-cell leukemia transcription factor 3 (PBX3), and cyclin-dependent kinase 6 (CDK6), and an increase in the expression of genes associated with differentiation, such as integrin alpha M (CD11b) and CD14. In the subset of *KMT2A*-rearranged AML, an overall response rate (ORR) of 59% and a CR [including CR with incomplete hematologic recovery (CRh)] of 33% were achieved. The median OS of all the population was 7 months [47]. Intriguingly, the analysis of patients with acquired resistance to menin inhibition revealed somatic mutations in *MEN1* after revumenib/menin treatment. These findings were confirmed in xenograft models and proved that a chromatin-targeting strategy exerts sufficient selection pressure to drive the evolution of escape mutants as a mechanism of therapeutic resistance [48].

### 5.2. Ziftomenib

Another ongoing phase I-II trial, KOMET-001 (NCT04067366), is investigating another oral menin inhibitor, ziftomenib (KO-539). In the 2022 update, 30 R/R AML patients were enrolled, of whom 24 presented an *NPM1* mutation or *KMT2A* rearrangements. The dose-finding study showed a 600 mg dose as feasible and effective, obtaining an ORR of 42%, a CR/CRh of 25%, and a CR/CR with incomplete hematologic recovery (CRi) of 33.3% [49]. 

### 5.3. Other Menin Inhibitors

75276617ALE1001 (NCT04811560) is an ongoing phase 1 trial investigating JNJ-75276617, an oral menin inhibitor. In the 2023 update, 56 patients with R/R AML were enrolled, 57% with *KMT2A* rearrangements. In the dose-finding study, among the eight patients treated at the highest dose (900 mg × 2/die), four obtained an ORR (two presented *KMT2A* rearrangements) [50]. In the last ASH annual meeting, the results of a phase I/II trial investigating the menin–MLL inhibitor DSP-5336 in R/R AML patients were presented; out of the six patients enrolled with R/R AML and *KMT2A* rearrangements, one patient achieved CRi, one patient achieved a morphologic leukemia-free state, and one achieved stable disease with the clearance of peripheral blasts, the recovery of peripheral counts, the resolution of leukemic gingival infiltration, and a reduction in bone marrow blasts from 85% to 31% [51].

## 6. Experimental Data of Combination Therapy

The rationale of the use of menin inhibitor paired with preclinical studies has shown the importance of potential drug partners.

Fleischmann and colleagues tested revumenib in combination with tamibarotene, an RAR alpha agonist, in cell lines and patient-derived AML cells. The authors observed a significant and synergistic pro-apoptotic effect in MV4-11 cells carrying translocation t(4;11), resulting in *KMT2A::AFF1* and OCI-AML3 cells (*NPM1*-mutated cells). Furthermore, MOLM13 cells carrying a cryptic insertion (11;9), resulting in *KMT2A-MLLT3* and OCI-AML3 cells, showed an increased expression of CD11b. Of note is that, in HL-60 cells (acute promyelocytic leukemia, not carrying *NPM1* mutations or *KMT2A*r), no apoptotic or differentiating effect was observed. Primary AML cells from five patients carrying *KMT2A*r were analyzed. Among these samples, revumenib–tamibarotene combination was effective in inducing apoptosis and in increasing CD11b expression in one patient-derived cell line [carrying t(11;19)], and in another one [carrying t(9;11)], CD11b expression increased without obvious apoptotic effects [52].

Ling et al. observed that AML cell lines characterized by *KMT2A*r (MV4-11, MOLM13, THP-1) were sensitive to venetoclax, a BCL2-inhibitor. This finding inspired the authors to test venetoclax in combination with MI-503. The combined therapy exerted a synergistic, selective pro-apoptotic effect in *KMT2A*r-AML cell lines. The treatment was also effective in reducing the proportion of leukemic cells and in prolonging survival in cell-derived MOLM13 xenografts. Transcriptomic analysis revealed that (a) the histone deacetylases 9 (HDAC9), but not other types of *HDACs*, was consistently down-regulated in *KMT2A*r-AML; (b) HDAC9 inhibition with the HDAC inhibitor TMP-269 impaired AML proliferation; and (c) *HDAC9* knockout did not enhance MI-503–venetoclax efficacy. Moreover, RNA expression analysis shed light on the mechanism of action of the combination therapy, which resulted in the inhibition of the hypoxia pathway. Finally, hypoxia-inducing experiments showed that MI-503–venetoclax down-regulated HDAC9 by suppressing *HIF-1A* [53].

In the same line, Ye and colleagues investigated the synergistic effect of chidamide (HDAC inhibitor) with MI-3, a menin inhibitor, showing a strong synergistic effect suppressing the growth of *KMT2A*r cells (MOLM-13, MV4-11). Colony-formation assays and flow cytometry revealed that chidamide interacts synergistically with MI-3 via the generation of reactive oxygen species (ROS) production and mitochondrial damage. The authors then further investigated the mechanism of action of the combination therapy, observing differently regulated genes associated with survival and cytokine signaling pathways in MOLM-13 cells treated with MI-3 and chidamide alone or in a combination regimen. Furthermore, the gene set enrichment analysis suggested that interference with the DNA repair machinery might contribute to the synergistic interaction between chidamide and MI-3. These findings were confirmed in MOLM13 cell xenograft models [54]. 

The potential of combinations with venetoclax was also explored by Fiskus and colleagues. The authors observed an inhibition of Bcl2 protein levels and Cdk6 following SNDX-50469 (menin inhibitor) treatment. In addition, they investigated the use of respective inhibitors (venetoclax and abemaciclib) in inhibiting the residual levels of these proteins, finding a strong synergistic activity of each drug with SNDX-50469 on both cell lines (MV4-11, MOLM13, OCI-AML3) and patient-derived AML cells expressing *KMT2A*r or *NPM1* mutation. The venetoclax–SNDX-50469 combination was also effective in xenograft mice bearing *KMT2A*r or *NPM1* mutation with mutated *FLT3* [55].

Aubrey and colleagues, using functional genetic screens, identified a key role of IKZF1/IKAROS, a transcription factor, in maintaining leukemia genes’ expression and repressing tumor suppressor genes in *KMT2A*r AML. Thus, the authors successfully tested immunomodulatory drugs (IMiDs), such as thalidomide and newer-generation lenalidomide, pomalidomide, and iberdomide, able to induce IKAROS degradation, in both *NPM1*-mutated and *KMT2A*r cell lines. The lenalidomide–VTP-50469 combination displayed synergy in MV411 and MOLM13 cell lines, with the response correlated to the potency of IKAROS degradation. The combination therapy was also confirmed to be effective in *KMT2A*r- and *NPM1*-mutated AML-patient-derived xenograft models, leading to rapid leukemia cell clearance and the differentiation of myelomonocytic lineage [56].

These findings have recently been paired with the research by Bourgeois et al., who successfully tested, in *KMT2A*r- and *NPM1*-mutated AML models, mezigdomide (cereblon E3 ligase modulator), which was able to degrade IKAROS more efficiently (increased depth, rate, and duration of protein degradation) than lenalidomide and iberdomide. Furthermore, the authors showed that the combination of mezigdomide with VTP-50469 increased survival and overcame the *MEN1* mutations responsible for resistance in patients receiving menin inhibitor monotherapy [57].

Thus, based on the promising data of early trials and in vitro and in vivo experiments on therapy combination, several clinical trials are currently investigating the association between menin inhibitors and chemotherapy [AUGMENT-102 (NCT05326516), KOMET-007 (NCT05735184)], as well as venetoclax and/or hypomethylating agents [SAVE (NCT05360160), NCT03013998, KOMET-007 (NCT05735184), NCT04752163]. Intriguingly, the gene expression profiles of *KMT2A-PTD* AML and of *HOXA* genes show similarities with *NPM1*-mutated and *KMT2A*-rearranged AML, suggesting a role of menin inhibitors in this subset of patients [58]. Table 2 summarizes several studies highlighting outcomes of patients with *KMT2A* rearrangements after treatment with pharmacologic agents and/or HSCT.

## 7. Conclusions

The analysis of *KMT2A* rearrangements in AML patients shows diverse prognostic outcomes based on age range. In the last few years, advances in biology have broadened our knowledge on the rearrangement partners and the molecular mechanisms of leukemogenesis in *KMT2A* anomalies of AML. The worldwide availability of next-generation sequencing and the introduction of alternative antileukemic treatment strategies (e.g., new drugs and broader indications for HSCT) in clinical practice have led to the identification of an intermediate-risk subtype, *KMT2A::MLLT3*, and adverse-risk ones [rearrangements different from t(9;11)] which have recently been added to the newest ELN risk stratification.

The promising results obtained by the first phase I trial investigating revumenib have prompted the design of new clinical trials with different molecules and drug combinations, possibly mimicking the path of other successful predecessors among smart drugs, such as all-trans retinoic acid and arsenic trioxide for acute promyelocytic leukemia [59] and FLT3 inhibitors for *FLT3* mutant AML [60], leading to a change in the paradigms of the treatment and prognosis of the disease. Novel menin inhibitors are being tested for KMT2A conditions.

## Figures and Tables

**Table 1:** *KMT2A* partner genes in acute myeloid leukemia.

Partner Gene	Chromosomal Location	Infant AML	Pediatric AML	Adult AML	% Total AML
*MLLT3*	(9p22)	28.4%	36.5%	24.4%	30.4%
*MLLT10*	(10p12.3)	27.4%	23.4%	9.9%	18.7%
*KMT2A-PTD*	(11q23.3)	0	1.5%	25.6%	10.7%
*ELL*	(19p13.1)	15.2%	6.8%	11.5%	10.1%
*AFDN*	(6q27)	1.5%	9.03%	10.2%	8.1%
*MLLT1*	(19p13.3)	1%	6.02%	3.6%	4.2%
*MLLT11*	(1q21.3)	6.59%	2.15%	0.9%	2.4%
*SEPTIN6*	(Xq24)	3.55%	2.15%	0.45%	1.7%
*MLLT6*	(17q12)	0	1.07%	2.72%	1.6%
*EPS15*	(1p32.3)	1.01%	1.07%	1.58%	1.3%
*SEPTIN9*	(17q25)	1.01%	1.29%	1.36%	1.3%
*CBL*	Del (11) (q23.3q23.3)	0	0	0.22%	0.89%
*AFF1*	(4q21)	1.5%	0.2%	1.1%	0.8%
*SEPTIN5*	(22q11.2)	1.01%	0.64%	0.45%	0.71%
*ABI1*	(10p11.2)	1.52%	1.07%	0	0.71%

Abbreviations: AML, acute myeloid leukemia. Partner genes were extracted from Meyer C. et al., Leukemia. 2023;37(5):988-1005 [1].

**Table 2:** Treatments in patients with *KMT2A* rearrangements.

Population	Drugs	*KMT2A*r Type	Outcome	Reference #
Adult AML	-cytarabine/daunorubicin-based induction chemotherapy ± autologous stem cell transplantation -decitabine ± bortezomib	t(9;11) (p22;q23) t(6;11) (q27;q23) t(11;19) (q23;p13.1) t(11;19) (q23;p13.3) t/ins(10;11) (p13;q23)	% CR (42-77) % 3-year OS (41-5)	[10]
	-venetoclax + HMA	t(9;11) (p22;q23) t(11;19) (q23;p13.1) t(6;11) (q23;q27) t(10;11) (p11.2;q23) t(4;11) (q21.3;q23) t(11;17) (q13;q23) t(11;11) (q13;q23) t/ins(10;11) (p13;q23)	% CR 83 median OS 11.05 months	[36]
	-allo-HSCT	t(6;11) (q27;q23) t(9;11) (p22:q23) t(11;19) (q23;p13.1) t(10;11) (p12;q23) t(1;11) (q21;q23)	% 3-year OS 59.1	[38]
	-intensively treated	KMT2A-PTD	% CR 64.5 median OS 24.4 months	[42]
Adult R/R AML	-venetoclax + HMA	t(9;11) (p22;q23) t(11;19) (q23;p13.1) t(6;11) (q23;q27) t(10;11) (p11.2;q23) t(4;11) (q21.3;q23) t(11;17) (q13;q23) t(11;11) (q13;q23) t/ins(10;11) (p13;q23)	% CR 17 median OS 6.05 months	[36]
Adult R/R AML-ALL-MPAL	-revumenib (SNDX-5613)	t(9;11) (p22:q23) t(4;11) (q21.3;q23) t(11;19) (q23;p13.1) t(6;11) (q27;q23) t(11;17) (q13;q23) Mut. NPM1	% CR 33	[47]
Pediatric AML	-intensively treated + GO	t(6;11) (q23;q27) t(10;11) (p11.2;q23) t(10;12) (p12;q23) t(4;11) (q21.3;q23) t(11;19) (q23;p13.3)	% CR 77 % 5-year OS 50	[34]
	-allo-HSCT	t(9;11) (p22:q23) t(11;19) (q23;p13.1) t(1;11) (q21;q23) t(6;11) (q27;q23) t(10;11) (p12;q23) t(4;11) (q21.3;q23) t(5;11) (q31;q23) t(7;11) (q22;q23) t(8;11) (q24;q23)	% 3-year OS 52.1	[39]

Abbreviations: AML, acute myeloid leukemia; MPAL, mixed-phenotype acute leukemia; ALL, acute lymphocytic leukemia; *KMT2A*r, *KMT2A* rearrangement; HSCT, hematopoietic stem cell transplantation; CR, complete response; R/R, refractory/relapsed; PTD, partial tandem duplication; OS, overall survival; GO, gemtuzumab ozogamicin.
