# Correction: Guarnera et al. *KMT2A* Rearrangements in Leukemias: Molecular Aspects and Therapeutic Perspectives. *Int. J. Mol. Sci.* 2024, *25*, 9023

**DOI:** 10.3390/ijms252111743

**Published:** 2024-11-01

**Authors:** Luca Guarnera, Matteo D’Addona, Carlos Bravo-Perez, Valeria Visconte

**Affiliations:** 1 Department of Translational Hematology & Oncology Research, Taussig Cancer Institute, Cleveland Clinic, Cleveland, OH 44114, USA; guarnel@ccf.org (L.G.); daddonm2@ccf.org (M.D.); bravoc2@ccf.org (C.B.-P.); 2 Department of Biomedicine and Prevention, Tor Vergata University of Rome, 00133 Rome, Italy; 3 Department of Hematology and Medical Oncology, Hospital Universitario Morales Meseguer, CIBERER—Instituto de Salud Carlos III, University of Murcia, IMIB-Pascual Parrilla, 30005 Murcia, Spain

There was an error in the original publication [1]. The oncogenic rearrangements in infant ALL are always somatic, not constitutive or inherited. As correctly indicated in the publication, there is a dramatic incidence of *KMT2A* rearrangements during infanthood, constituting the driver genetic alteration in 80% of acute leukemias during this period. *KMT2A*-rearranged ALL normally has a poor prognosis. In this manuscript, the term “germline *KMT2A* rearrangement” is incorrect and needs clarification. Instead, it was intended to specify that 20% of the infants with ALL which were not *KMT2A*-rearranged, also known as *KMT2A*-germline since the gene remained in its unaltered (germline) configuration, were found to carry rearrangements in other oncogenes. Similarly, the term “germline *KMT2A::NUTM1*” is not correct. Instead, in the *KMT2A*-germline cases, genetic analysis showed an enrichment in the *NUTM1* and *PAX5* rearrangements, which were fused to partners not including *KMT2A*. Genetic correlation studies revealed that *NUTM1* rearrangements have a favorable prognosis (PFS of 75–95%), while *PAX5* and other gene rearrangements have a high risk of relapse.

A correction has been made to “Chapter 3.1. Incidence, Clinical Features, and Prognosis in Infancy and Childhood”, first paragraph, as follows:

There is a dramatic incidence of *KMT2A* rearrangements in infants, constituting the driver genetic alteration in 80% of the acute leukemias during this period. Most of them are B-ALL (80–90%), followed by T-ALL, AML (up to 5–10% each), and other disease subtypes (<5%) [21]. Molecularly, the most frequent fusion partners of *KMT2A* are *AFF1*, *MLLT1*, and *MLLT3* [1]. Infant-onset B-ALL with *KMT2A* rearrangement normally exerts a very immature phenotype (pro-B), with the frequent aberrant co-expression of myeloid markers. Patients normally present with hyperleukocytosis and extramedullary disease with central nervous system involvement and have a poor prognosis [22]. The other 20% of infant ALL cases that were not *KMT2A*-rearranged, also known as *KMT2A*-germline since the gene remained in its unaltered (germline) configuration, were found to carry somatic rearrangements in other oncogenes. Genetic analysis showed an enrichment in the *NUTM1* and *PAX5* rearrangements. Clinically, *KMT2A*-germline cases are normally B-ALLs with a more mature immunophenotype. Genetic correlation studies have revealed that *NUTM1* rearrangements have an unusual favorable prognosis as compared to the rest of the infant ALL (PFS of 75–95%), while *PAX5* and other gene rearrangements have a high risk of relapse [23–26].

The authors state that the scientific conclusions are unaffected. This correction was approved by the Academic Editor. The original publication has also been updated.
